# Genetic Diversity, Population Structure, and Historical Gene Flow Patterns of Nine Indigenous Greek Sheep Breeds

**DOI:** 10.3390/biology14070845

**Published:** 2025-07-10

**Authors:** Sofia Michailidou, Maria Kyritsi, Eleftherios Pavlou, Antiopi Tsoureki, Anagnostis Argiriou

**Affiliations:** 1 Institute of Applied Biosciences, Centre for Research and Technology Hellas, 57001 Thessaloniki, Greece; kyritsimaria@certh.gr (M.K.); elefpavlou@gmail.com (E.P.); adatsoureki@certh.gr (A.T.); argiriou@certh.gr (A.A.); 2 Department of Food Science and Nutrition, University of the Aegean, 81400 Myrina, Greece

**Keywords:** sheep, Greek breeds, population structure, admixture, genetic diversity, heterozygosity, homozygosity, gene flow

## Abstract

Ιn this study, we evaluated the genetic resources of nine Greek sheep breeds. The genotyping data of 292 animals were acquired from Illumina’s OvineSNP50 Genotyping BeadChip. The genetic diversity and inbreeding levels were evaluated using the observed and expected heterozygosity indices, the F_IS_ inbreeding coefficient, and runs of homozygosity (ROH). The genetic differentiation of breeds was assessed using the F_ST_ index, whereas their population structure was analyzed using admixture and principal components analysis (PCA). Historical recombination patterns and genetic drift were evaluated based on linkage disequilibrium, effective population sizes, and gene flow analysis to reveal migration patterns. PCA revealed distinct clusters mostly separating mountainous, insular, and lowland breeds. The F_ST_ value was the lowest between Serres and Karagouniko breeds (0.050). Admixture analysis revealed a genetic substructure for Serres and Kalarritiko breeds, while Chios, followed by Katsika, demonstrated the highest within-breed genetic uniformity. ROH analysis revealed low levels of inbreeding for all breeds. Genetic introgression from both Anatolia and Eastern Europe has been evidenced for Greek sheep breeds. The results also revealed that Greek sheep breeds maintain adequate levels of genetic diversity, without signs of excessive inbreeding, and can serve as valuable resources for the conservation of local biodiversity.

## Simple Summary

The rapid development of biological sciences in the 21st century opened up new horizons and perspectives in livestock breeding, which is now governed by new rules based on information related to their genetic background. Greece, a country that is traditionally linked to sheep farming from ancient times, could not follow modern practices applied in many European countries, such as genetic improvement and the application of biotechnological breeding techniques, and hence, fell behind in the creation of sheep breeds that could serve as nuclei for breeding strategies with the goal of improved performance and increased resilience to harsh environments. In this study, we analyzed the genetic background of the most numerous or important Greek sheep breeds to evaluate the local genetic resources and aid in actions either for breed preservation or genetic improvement. In addition, through gene flow analysis, we provide insights into the migration waves and domestication of sheep in the Mediterranean basin. Our findings form a basis on which genetic improvement and selection schemes can operate to produce a breeding stock with the desired origin and traits.

## 1. Introduction

In Greece, sheep breed formation took place in the distant past, shaping the core of the current genetic resources. Based on genetic studies employing single-nucleotide polymorphism (SNP) [1] and mitochondrial DNA (mtDNA) data [2], it is believed that sheep were introduced to Europe around 8000–9000 years BP (Before Present) following two routes: (i) through Anatolia (from the cradle of their domestication center towards the west through Turkey, Cyprus, and Greece) and (ii) through the Balkans (following the Danubian corridor), being spread further along the coastline of the Mediterranean or inland Europe to reach the coasts of northwestern Europe [3]. Thereafter, distinct characteristics developed in each sheep breed as a result of their coexistence with humans, based on the lineage followed and transhumance activities [4,5]. Greece’s geographic location at the intersection of three continents has historically made it a crossroads for different migration routes. As a result, it is believed that breeds from both the Levant region and the Northern Balkans have influenced Greek sheep, contributing to the formation of populations and breeds with significant differences in morphological characteristics. Nowadays, most of the mountainous Greek sheep breeds belong to the Zackel type, like the Boutsko, Kalarritiko, Katsika, and Thraki breeds. In addition, sheep of the Ruda type, like Pelagonia and Serres breeds, or semi-fat-tailed breeds, like Chios and Lesvos [6], are also found. Except for the Chios breed, which is the only one that has been subjected to long-term national programs for breeding and performance recording, most Greek breeds are unimproved, comprising mostly low-demanding animals that produce adequately and are well adapted to harsh environments.

The importance of preserving local genetic resources is a global concept supported by several organizations like the Food and Agriculture Organization (FAO) [7], the International Union for Conservation of Nature (IUCN) [8], and the Rare Breeds Survival Trust (RBST) [9]. Especially for the European Union (EU), the Reference Centre for Endangered Animal Breeds (EURC-EAB) has published guidelines on the preservation of livestock’s genetic diversity, particularly for endangered breeds, following Article 29(2) of Regulation (EU) 2016/1012 [10]. Evolutionary scientists support the idea that if adequate levels of within-species genetic diversity are not conserved, tremendous effects will take place regarding both ecological and economic aspects [11]. Therefore, the conservation of autochthonous breeds should be a priority in animal science and breeding, as these individuals may hold the key to overcoming challenges related to performance and survival in changing environments. In addition, the success of selective breeding schemes and reproductive strategies relies on the evaluation of individuals’ breeding values, which can be accurately assessed using genome-wide genetic markers.

Greece has a large number of recognized distinct sheep breeds that exhibit large phenotypic and morphological diversity (Figure S1, Table S1). Many of them are reared in small populations, putting their survival at risk, while several of them are already threatened with extinction. Although levels of genetic diversity are considered an important factor influencing a population’s long-term potential for survival, little research has been conducted on Greek breeds using genome-wide data. Previous studies reported high levels of genetic diversity for the Chios, Karagouniko, Boutsko, and Lesvos breeds [12,13]. Nevertheless, there are no studies on the genetic resources of breeds not so frequently or intensively reared, such as Kalarritiko, Katsika, Thraki, Pelagonia, or Serres. Kalarritiko is a small-sized, dual-purpose breed, as it demonstrates excellent quality both in its milk and meat composition. Its population is estimated to be around 6000 individuals, reared following the extensive breeding system in the mountainous area of Epirus, mainly involving transhumance activities. The Safeguard for Agricultural Varieties in Europe (SAVE) foundation has reported that the Kalarritiko breed was imported from southern Italy 250–300 years ago, and is probably a descendant of the Comisana breed of Sicily [14]. Nowadays, it is considered that the Kalarritiko and Boutsko breeds are closely related and are also referred to under the broader term ‘Orino of Epirus’ (“Orino” meaning “mountainous”). The Pelagonia and Katsika breeds comprise medium-sized ewes of low milk performance. Both breeds are at risk and could become endangered if current practices in sheep breeding schemes continue; about 1000 and 1600 purebred animals are considered to exist for the two breeds, respectively. Especially for Katsika, there are only a few farms breeding purebreds, a fact that raises concerns about the long-term viability of this breed. Thraki’s purebreds are estimated to number fewer than 1000 individuals, reared by the Thracian Pomak communities in the mountainous region of northwest Rodopi municipality under the extensive breeding system. These small-sized animals are well adapted to the area’s climatic conditions, exploiting the difficult-to-access regions at a high rate [15]. The Serres breed numbers about 6000 animals and is reared both for milk and meat, as the breed’s animals produce large lambs and, consequently, carcasses. Serres sheep are well adapted to harsh mountainous and semi-mountainous regions, where they exploit most of the available pastures. Karagouniko is a typical lowland, thin-tailed, and mixed-wool sheep breed originating from Central Greece and is distinguished for its relatively high milk production (180–250 kg) and adaptation to marginal conditions [16]. It has been involved in many breeding schemes with other autochthonous breeds like Chios; thus, crossbred animals are often found within flocks. Chios is an insular, highly productive dairy breed in terms of milk yield and prolificacy. It is a semi-fat-tailed breed with a fine wool quality [17], originating from the island of Chios. Chios is the only Greek breed that has been included in national reproductive breeding programs focused on performance recordings and genetic improvement. Finally, Lesvos is another insular, medium-sized sheep breed that originates from the homonymous island of Lesvos in the Aegean Sea. Numerous purebreds of this breed are bred (260,000 animals) on the islands of the North Aegean Sea and in Northern Greece. Lesvos sheep exhibit good milk performance and demonstrate good climate resilience in mountainous and semi-mountainous regions, exploiting poor pastures very well [18]. Overall, indigenous Greek sheep breeds serve as valuable genetic resources, offering unique traits that are crucial for ensuring long-term sustainable livestock farming and adaptation to different environments [19,20]. For instance, although Chios sheep are highly productive under intensive breeding systems (in terms of milk yield and reproductive performance), they are not suitable for extensive breeding systems due to their susceptibility to diseases and their limited ability to perform well under harsh or extreme environmental conditions [21]. Greece has the benefit of rearing sheep breeds that can exploit limited or poor pastures, while showing resilience in extreme climates. Hence, their ability to thrive under such challenging conditions highlights their genetic resources’ importance in the context of climate crisis, as future sustainable livestock systems will depend on animals capable of coping with environmental stressors.

Concerning breed formation in Europe, Ciani et al. (2020) highlighted that Greek sheep breeds acted as a barrier between Asian and European breeds [1]. Indeed, Greece has remained a blind spot on the evolutionary map of sheep migration from the Fertile Crescent to Western Europe, as no historical gene flow patterns have been estimated thus far. Hence, with this work, we unveil the sheep migration routes and their domestication in Europe, including their passage through the Greek territory, by analyzing the historical gene flow combined with sheep breeds from other continents as well (Asia, Oceania, and Africa). Our results give insights into the genetic introgression of Greek insular breeds with breeds from Anatolia and the transfer of genetic material from breeds of Eastern Europe and the Balkans to lowland Greek sheep breeds. In addition, in this work, we evaluate the levels of genetic differentiation of nine Greek sheep breeds and estimate their genetic diversity levels, since the available genetic resources can ensure livestock sustainability and are a key factor in the country’s economic development.

## 2. Materials and Methods

### 2.1. Animal Sampling and DNA Extraction

All applicable international, national, and/or institutional guidelines for the care and use of animals were followed. Blood sampling was conducted by veterinarians or under veterinarians’ supervision for routine veterinary care, with the written consent of the breeders. In total, 292 blood samples from ewes originating from 12 farms (populations) in Northern Greece were collected. These samples were assigned to nine breeds based on the available pedigree, morphology, and breeding records: Boutsko (N = 29), Chios (N = 24), Kalarritiko (N = 46), Karagouniko (N = 26), Katsika (N = 20), Lesvos (N = 39), Pelagonia (N = 36), Serres (N = 42), and Thraki (N = 30). Information on the farm locations and breeding systems for each breed is presented in Table S2. Moreover, animals belonging to the Assaf (N = 25) and Charollais (N = 20) breeds, reared in the Greek territory, were also genotyped and incorporated in the population structure and gene flow analyses for comparison purposes.

For genetic analyses, 5 mL of blood were collected from the jugular vein of each ewe in tubes containing spray-dried K2EDTA as an anticoagulant and stored at 4 °C until further processing in the laboratory. DNA extraction was performed using the Nucleospin Blood Quick Pure kit (Macherey-Nagel, Düren, Germany) according to the manufacturer’s instructions. DNA integrity and quantity were assessed by electrophoresis on a 0.8% TAE agarose gel and using the Qubit^®^ dsDNA BR assay kit (Qubit 4.0 Fluorometer; Thermo Fisher Scientific, Waltham, MA, USA), respectively.

### 2.2. Genotyping and Data Quality Filtering

For genotyping, 400 ng of high-quality DNA was dried in 1 mM of Tris-EDTA and genotyping was performed at GeneSeek (Neogen Corporation, Ayr, UK) on Illumina’s iScan platform. Animals were genotyped using different versions of Illumina’s OvineSNP50 Genotyping BeadChip; 190 animals were genotyped using version 2, comprising 53,516 SNPs, and 102 animals were genotyped using version 3, featuring 63,840 SNPs. These different datasets were merged, and only common SNPs among them were retained for further analysis.

SNP quality filtering was performed in PLINK v1.90 [22]. Specifically, variants with a minor allele frequency (MAF) < 1%, call rate < 0.98, and Hardy–Weinberg equilibrium (HWE) *p*-value ≤ 1 × 10^−6^ were filtered out. Sex-linked SNPs and those with an unknown genomic position were also removed. In addition, for principal components (PCA) and gene flow analyses, SNP datasets were linkage disequilibrium (LD) pruned using the “--indep-pairwise” option in PLINK v1.90 with a window size of 50 SNPs, a step size of 5 SNPs, and *r*^2^ > 0.2. For the detection of runs of homozygosity (ROH), quality filtering did not include filtering for MAF and LD, according to Meyermans et al. (2020) [23]. Sample filtering was conducted for individuals with missing call rates exceeding 10%.

### 2.3. Population Structure, Gene Flow, and Admixture Analyses

Evaluation of the population structure of Greek sheep breeds was conducted using SNP datasets from foreign and cosmopolitan breeds reared worldwide, to examine their genetic clustering. These breeds (or populations) included ewes from Europe (N = 51), Oceania (N = 2) [24,25,26], Asia (N = 23) [27,28], and Africa (N = 4) [29]. The selection of breeds was based on their region of origin in relation to the known migration routes, from their domestication center to Europe, encompassing both the Danubian corridor and the Mediterranean basin. Thus, including the Greek sheep breeds, 89 breeds/populations, counting 1661 individuals in total, were analyzed (Table S3). Data integration and filtering were performed as previously described for the Greek sheep-only dataset.

Principal component analysis (PCA) was conducted on filtered, LD-pruned SNPs using PLINK v1.90 for the calculation of eigenvalues and eigenvectors and visualized in R (v4.3.3) [30] using the ggplot2 (v3.5.2) [31] and ggrepel (v0.9.6) [32] R packages. PCA was performed both on the worldwide dataset, which included all 89 breeds, and on the dataset of only Greek sheep to better visualize clustering patterns across the nine Greek breeds.

Historical gene flow analysis was performed using the TreeMix (v1.12) software [33], implementing a pipeline based on the scripts available on GitHub [34], available online: https://github.com/carolindahms/TreeMix (accessed on 8 April 2025). For gene flow analysis, we used a reduced dataset that was based on PCA results, retaining representative breeds from each country and continent. Thus, the gene flow dataset comprised 54 breeds. The cluster-stratified frequencies were estimated from the binary pedigree dataset using PLINK v1.90 [22] with the parameters “--freq” and “--within”. The input file for the TreeMix analysis was generated using the “plink2treemix.py” script provided by the treemix package. A consensus unrooted maximum likelihood (ML) tree was constructed using the “consense” program of the PHYLIP package (v3.696) [35] from 100 bootstrap replicates, with a SNP block size of 200 consecutive SNPs. Migration events were considered in the range of 1 to 15 (option “-m”), with five iterations performed for each value of m. The optimal number of migration edges was inferred by assessing multiple linear models with the OptM (v0.1.8) [36] R package. Then, the final independent TreeMix runs with the optimal m set to seven were performed with the following settings: SNP block size set to 200 (option “-k”), Asian Mouflon as the outgroup (option “-root”), 100 bootstrap replicates (option “-nboot”), and five (option “-migrep”) iterations to test each number of m. A detailed ML tree annotated with bootstrap values and migration edges was generated using the “treemix.bootstrap()” function of the BITE (v2.1.2) package in R [37].

Admixture analysis was performed only on Greek breeds to test for the population substructure, assuming a number of subpopulations (K) from 2 to 12, using the ADMIXTURE (v1.3) ancestry estimation software [38]. Admixture results were plotted in circular form using the BITE (v2.1) R package [37]. To identify the true number of breeds or populations, we used the model with the lowest cross-validation (CV) error.

### 2.4. Genetic Diversity Indices

To evaluate the genetic diversity within each Greek breed, the observed (Ho) and expected (He) heterozygosities were estimated using the ARLEQUIN (v3.5.2.2) software [39] by applying a correction step on the number of usable SNPs proposed by Colli et al. (2018) [40]. The same software was also used for estimating nucleotide diversity (π) and the number of within-breed polymorphic SNPs.

### 2.5. Genetic Differentiation

The levels of genetic differentiation among Greek breeds were assessed using Wright’s pairwise F_ST_ (Subpopulation within Total population) value. Coancestry coefficients were computed by adopting 1000 permutations for the Mantel test, using the ARLEQUIN (v3.5.2.2) software [39]. A *p*-value threshold of 0.05 was adopted to determine significant genetic differentiation.

### 2.6. Runs of Homozygosity and Inbreeding Levels

The patterns of inbreeding within each breed were assessed through ROH detection, as proposed by Meyermans et al. (2020) [23], without applying MAF and LD filtering on the data. ROH was calculated for each autosome per breed using the “homozyg” function in PLINK v1.90. Furthermore, ROH was defined if the following criteria were met: (i) the minimum ROH length should be 1 Mb to remove very short segments; (ii) each ROH should contain at least 50 SNPs; (iii) the minimum SNP density should be 1 SNP per 70 kb (kilobase); (iv) each scanning window should contain at least 50 SNPs; (v) the maximum gap allowed between consecutive SNPs in ROH should be 100 kb; (vi) only 5 missing SNPs are allowed per ROH; (vii) the maximum heterozygous SNPs allowed per ROH should equal 1; and (viii) the scanning window threshold should be set at 0.05. ROHs were plotted per breed as the total number per animal against the total length of ROHs.

To further investigate inbreeding levels, three different inbreeding coefficients were calculated: (i) Wright’s inbreeding coefficient F_IS_ (Individual within Subpopulation), which measures the excess of homozygotes or heterozygote deficiency within breeds, using PLINK v1.90; (ii) the F_GRM_ inbreeding coefficient per breed, calculated using the GCTA program [41] after constructing the Genetic Relationship Matrix (GRM); and (iii) the F_ROH_ coefficient, calculated separately for each animal as the sum of ROH lengths, divided by the total length of the autosomal genome (in kb) covered by SNPs, as proposed by McQuillan et al. (2008) [42].

### 2.7. Linkage Disequilibrium and Effective Population Sizes

The LD between all SNP pairs per breed was analyzed to examine the non-random association of alleles using PLINK v1.90. LD was calculated based on the squared correlation coefficient (*r*^2^) by setting a threshold of 0.2 and a physical distance (LD window) spanning from 0.001 to 1 Mb. The LD decay plot based on pairwise *r*^2^ was constructed by determining the nonlinear least squares fit line using the “nls” function in R. The average *r*^2^ per breed was estimated as the arithmetic mean of all *r*^2^.

The effective population size (Ne) for the past 100 and 1000 generations was calculated separately per breed using the SNeP (v1.1) program [43]. SNeP was run considering the LD decay across the genome, and the recombination rate was adjusted using the “svedf” function, which controls Ne estimates by reducing bias due to small sample sizes. Ne sizes were obtained for the last 1000 generations and relative diversity trends were plotted in R for the last 100 and 1000 generations.

## 3. Results

### 3.1. SNP Quality Control

After merging the data from the two different versions of the OvineSNP50K, a total of 53,445 SNPs were retained across all datasets. Quality control excluded 1101 SNPs due to MAF filtering, 43 SNPs due to a low call rate, and 1165 SNPs due to HWE filtering. Additionally, 1608 SNPs were sex-linked or without chromosomal coordinates and thus were discarded. Overall, quality filtering resulted in a dataset comprising 49,528 SNPs and 292 animals (no sample was excluded due to low genotypic call rate). For gene flow analysis and PCA, the quality-filtered and LD-pruned dataset, incorporating the foreign and cosmopolitan breeds, included 31,617 SNPs, whereas the respective Greek dataset contained 39,106 SNPs. For ROH analysis, the filtered dataset included 50,728 SNPs, since the MAF and LD pruning filtering steps were omitted to improve ROH detection. In all cases, the total genotyping rate was consistently greater than 0.994, indicating the high quality of the analyzed datasets.

### 3.2. Genetic Structure and Historical Gene Flow Patterns Compared to Foreign Breeds

**Figure 1:**
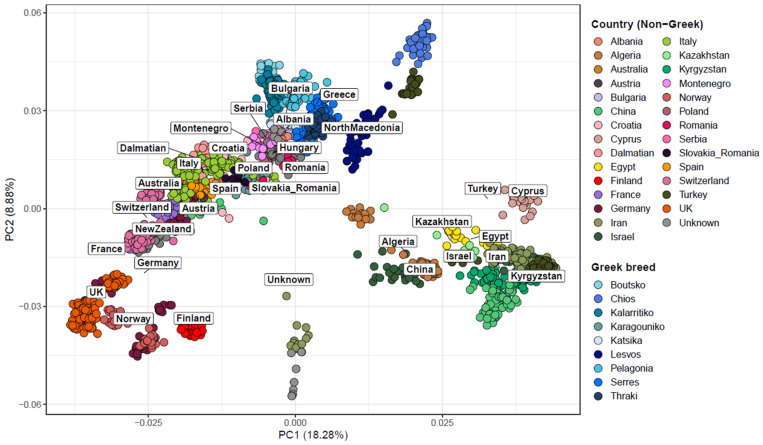
Principal component analysis (PCA) of the first two components in 1661 sheep samples originating from 89 breeds worldwide. Each dot represents a sample, and the samples are colored based on the country of origin. The wild Urial and wild Argali populations are denoted as “wild”. Greek sheep breeds are presented in varying shades of blue for better visual distinction.

Population structure analysis of Greek sheep breeds was performed, in combination with 80 breeds from other countries, to evaluate their genetic relatedness and investigate their genetic relationship in a global context. PCA revealed three major clusters: the first comprising wild sheep (Urial—*Ovis vignei* and Argali—*O. ammon*) and Asian Mouflon (*O. orientalis*), the second consisting of Asian and African breeds (*O. aries*), and the third of European and Oceanian breeds (*O. aries*) (Figure 1). The latter cluster demonstrated a wide, loose structure, which extended from the Greek breeds at one end to breeds originating from the UK and Northern Europe at the other. For the Greek breeds, it was evidenced that lowland breeds were found in close proximity to breeds from Eastern Europe and the Balkans, whereas insular breeds of the Aegean Sea (Chios and Lesvos) were closer to the Turkish Sakiz breed. In particular, Chios and Lesvos breeds were found to be genetically more differentiated from the rest of the Greek sheep breeds. Overall, the first two principal components (PC1 and PC2) explained a substantial proportion of the total genetic variation, with PC1 accounting for 18.28% and PC2 for 8.88%.

**Figure 2:**
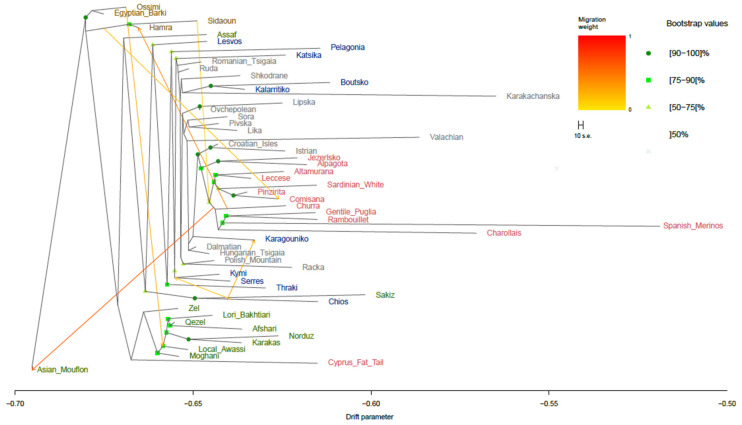
TreeMix analysis for the 54 sheep populations with seven migration events. Only bootstrap values above 50% are shown. Migration edges are colored according to their migration weight. Breeds are colored according to their geographical origin (brown for Africa, green for Asia, grey for the Balkans and Eastern Europe, blue for Greece, and red for Southern and Western Europe). The drift parameter is plotted on a log10 scale for the better visualization of branch separation.

Based on the PCA results, gene flow patterns were evaluated in a reduced dataset, excluding breeds and populations that clustered far from the Greek breeds. However, breeds from Asia and North Africa were retained to capture the potential migration waves. Hence, 54 breeds were kept for gene flow analysis, revealing the migration scenarios across sheep breeds (Figure 2 and Figure S2). The model with the seven migration edges was chosen to better capture historically plausible gene flow events among sheep breeds, reflecting the extensive mobility and different management practices of domesticated sheep. The ML tree inferred similar groupings of breeds to those found in the PCA, with most of the nodes supported by bootstrap values higher than 75%. From the seven major migration waves identified, two involved Greek breeds. The first one involved a medium-weighted edge that linked Chios to the Karagouniko breed, whereas the second medium-weighted edge informed on the contribution of genes from Chios to the Kymi and Serres breeds. A clear separation of Asian, African, and European breeds was observed, except for the Sakiz breed, which formed a separate clade with the Chios breed. This clade is most closely related to the ancestor of the Lesvos breed. Furthermore, the shared ancestor of Chios and Lesvos originates from the same ancestral lineage as the Assaf breed, highlighting the genetic proximity of the two Greek insular breeds with Assaf. On the contrary, the remaining mainland Greek breeds were located in various clades, separate from the insular breeds. Specifically, Katsika was placed in a joint cluster with the Romanian Tsigaia and Ruda sheep from Hungary. Similarly, Karagouniko was also grouped with breeds from the Northern Balkans; the Valachian, Dalmatian, and Hungarian Tsigaia sheep were the closest relatives. In addition, the Kalarritiko and Boutsko breeds were clustered within the same subtree with breeds from Albania (Shkodrane) and Bulgaria (Karakachanska).

### 3.3. Population Structure, Genetic Differentiation, and Admixture for Greek Breeds

**Figure 3:**
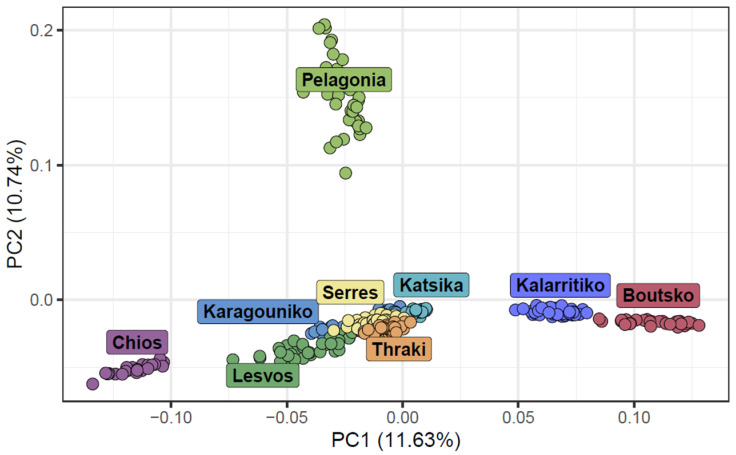
Principal component analysis (PCA) of the first two components for the nine Greek sheep breeds. Each breed is represented by a different color.

To further investigate the genetic structure and differentiation among indigenous Greek sheep breeds, we performed a PCA focusing solely on these breeds. The population structure based on PCA revealed distinct clusters with clear boundaries for most of the studied Greek sheep breeds. However, some breeds were found to overlap. These breeds included lowland breeds, like Karagouniko and Serres, as well as mountainous breeds, like Katsika and Thraki. Kalarritiko and Boutsko were found in close proximity, as expected, considering the common origin and phenotype of these two breeds. The insular breeds, Lesvos and Chios, were placed at a great distance from the mountainous breeds, with Chios, in particular, being located in a tight cluster away from all other breeds. The Pelagonia breed exhibited the most dispersed clustering pattern, and, in addition, it was placed far from the other breeds, suggesting that this breed had higher levels of genetic differentiation compared to the others. The first two principal components (PC1 and PC2) accounted for a significant portion of the total genetic variation, with PC1 explaining 11.63% and PC2 explaining 10.74% (Figure 3).

The genetic differentiation of the breeds and their level of relatedness, as measured by pairwise F_ST_ values, indicated the close proximity of Serres with the Karagouniko breed, demonstrating a value of 0.050. Small genetic differentiation was also observed between the Serres and Lesvos breeds, as well as the Serres and Kalarritiko breeds (0.055 for both pairs). On the contrary, the highest F_ST_ values were obtained for Chios and Boutsko (0.157), suggesting high genetic differentiation between them, followed by the Chios and Pelagonia pair (0.139). Overall, the genetic differentiation among Greek sheep breeds was moderate (mean F_ST_ values of 0.089), presented in detail for each pair in Table 1.

**Figure 4:**
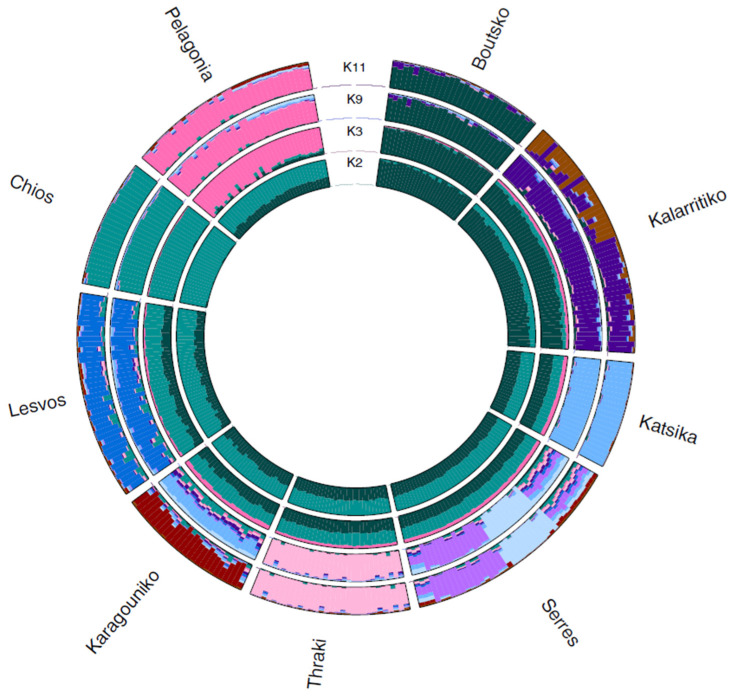
Circular representation of admixture analysis at the numbers of subpopulations (K), K = 2, 3, 9, and 11 (best K) for the 292 animals of the nine Greek sheep breeds. Each color represents a distinct source ancestry, while each individual is represented by a segment showing the proportion of ancestry derived from each source.

Admixture analysis was also conducted for Greek sheep breeds to investigate the within-breed population substructure. The results showed that the optimal K was 11, as it demonstrated the lowest cross-validation error (0.576), revealing the population stratification for some breeds. Most breeds displayed a uniform, distinct ancestry with no admixture, while others exhibited significant genetic mixing, suggesting historical interbreeding or a shared ancestral background (Figure 4). At K = 2, the first breed that was differentiated was Chios, which also maintained the most uniform profile for all subsequent Ks analyzed. Following Chios, the Pelagonia breed was differentiated at K = 3. A population substructure was observed at K = 9 for Serres and at K = 11 for the Kalarritiko breed. For both breeds, the admixed genetic profile and population substructure were linked to individual farms, reflecting potential influence of farmer-driven selection practices or past crossbreeding.

### 3.4. Within Breed Genetic Diversity and Inbreeding Levels

The calculation of genetic diversity indices was performed to assess the levels of genetic variation within breeds. The results showed that the percentage of polymorphic loci was high for all breeds, ranging from 93.17% (Chios) to 98.81% (Serres), suggesting a diverse gene pool for the Greek breeds (Table 2). The within-breed genetic diversity levels, as measured by observed (Ho) and expected (He) heterozygosity indices, revealed that Greek sheep breeds maintain adequate levels of genetic diversity. The Chios breed had the lowest Ho and He values (0.326 and 0.344, respectively). Following Chios, Pelagonia and Boutsko had the lowest Ho (0.348 and 0.352, respectively) and He (0.355 and 0.353, respectively) levels. On the contrary, the highest levels of genetic heterozygosity were observed for the Karagouniko breed (0.379 Ho and 0.373 He), which is attributed to the absence of controlled breeding schemes for this breed thus far. The same trend concerning the levels of genetic diversity was observed when investigating the within-breed nucleotide diversity (π); Chios presented the lowest value (0.327), whereas Karagouniko presented the highest (0.371). However, it should be noted that all breeds demonstrated high levels of genetic diversity, indicative of low inbreeding levels and an absence of selective pressure.

**Figure 5:**
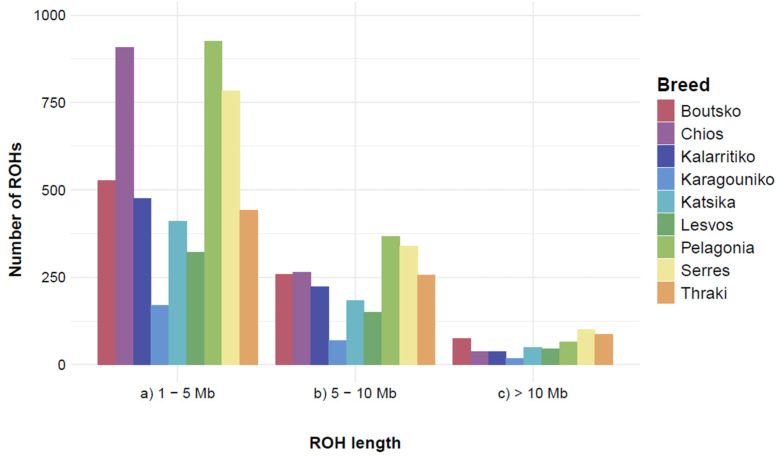
Runs of homozygosity (ROHs) classification per breed, according to their length. ROHs were classified into three length categories ((a) short: 1–5 Mb, (b) medium: 5–10 Mb, and (c) long: >10 Mb ROHs). Mb: Megabase.

Inbreeding levels, as measured by ROH analysis, revealed that the Greek breeds present low levels of autozygosity, since a small proportion of their genome was found homozygous by descent. Based on the thresholds applied, a total of 7582 ROHs were identified in 282 of the 292 animals. Pelagonia presented the highest number of ROHs (N = 1356), followed by Serres (N = 1223) and Chios (N = 1210). On the contrary, Karagouniko had the smallest number of ROHs (N = 256) (Figure S3). On average, Chios had the highest number of ROHs per individual (N = 50.4), followed by Pelagonia (N = 37.7), and Katsika (N = 32.2), whereas Karagouniko had the lowest (N = 9.85). The largest ROHs were 38.72 Mb, 24.54 Mb, and 24.34 Mb, and were identified in the Thraki, Pelagonia, and Lesvos breeds, respectively. However, the mean ROH length was similar among breeds, ranging from 4.27 Mb (Chios) to 5.66 Mb (Thraki). The three largest ROHs were all found on chromosome 2. Yet, concerning the percentage of chromosome length covered by ROHs, a trend suggesting an inverse relationship between chromosome size and percentage covered by ROH was observed, with the highest percentages reported to have a decreasing chromosome length (Figure S4). Chromosome 25 had the highest percentage of ROHs (20.19%), followed by chromosome 26 (18.98%) and chromosome 19 (18.05%), while chromosomes 1, 2, and 3 had the lowest (7.99%, 8.36%, and 8.75%, respectively). After categorizing ROHs according to their size, 4960 short ROHs (1–5 Mb), 2108 medium-sized ROHs (5–10 Mb), and 514 long ROHs (over 10 Mb) were identified (Figure 5). All breeds exhibited the most ROH segments in the short-length category (1–5 Mb), with the reported numbers decreasing with increasing length category.

Inbreeding levels were also estimated using the F_IS_, F_GRM_, and F_ROH_ coefficients; in all cases, the Chios breed demonstrated the lowest levels of heterozygosity, whereas Karagouniko demonstrated the highest (Table 3). In particular, for Wright’s F_IS_ inbreeding coefficient, all breeds presented positive, although very close to zero, values, indicating a slight excess of homozygotes in the studied populations. F_IS_ values ranged from 0.011 (Karagouniko) to 0.148 (Chios). The F_GRM_ inbreeding coefficient revealed a higher genetic relatedness of Chios individual animals (0.126) compared to the other breeds. Likewise, Katsika presented increased within-breed genetic similarity (0.083), whereas Lesvos (0.038) and Karagouniko (0.036) had the lowest F_GRM_ values. Inbreeding levels based on F_ROH_ revealed higher levels of autozygosity in the Chios breed (F_ROH_ = 0.082) compared to the others. However, across breeds, differences in the mean proportion of the autosomal length were moderate. Large ROHs (over 10 Mb) presented the highest F_ROH_ values among the different length categories (mean values for all breeds: F_ROH_1-5_ = 0.001, F_ROH_5-10_ = 0.003, F_ROH_>10_ = 0.056). Overall, based on the inbreeding coefficients examined (F_IS_, F_ROH_, and F_GRM_), similar levels of autozygosity were found across breeds, with Chios and Karagouniko being the least and the most genetically diverse, respectively.

### 3.5. Linkage Disequilibrium and Effective Population Size

**Figure 6:**
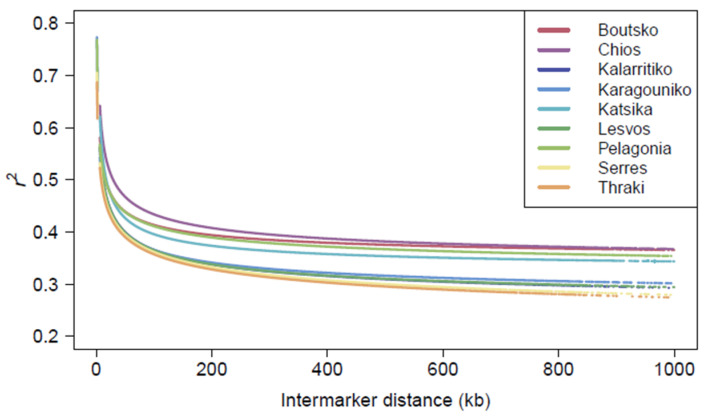
Linkage disequilibrium (LD) decay over autosomal chromosomes per breed, based on *r*^2^ and pairwise inter-marker distance in kb.

The non-random association of alleles at different loci was assessed with the pairwise *r*^2^ in the 49,528 autosomal SNPs for each breed separately. A comparison of the LD patterns across sheep breeds revealed a stratification of breeds related to their genetic makeup (Figure 6). The highest average *r*^2^ value was obtained for the Chios breed (0.418), followed by the Boutsko (0.400), Pelagonia (0.398), and Katsika (0.383) breeds. On the contrary, the lowest average *r*^2^ values were obtained for the Serres (0.354), Lesvos (0.356), Thraki (0.357), Karagouniko (0.357), and Kalarritiko (0.359) breeds. Overall, the trend of LD decay was quite similar across breeds, with the most rapid decline observed over the first 1000 kb. As expected, it was shown that as the distance between SNP markers increased, the *r*^2^ values gradually decreased.

The average inter-marker distance for Greek sheep breeds was 218,523 kb, ranging from 185,931 kb for Thraki to 237,560 kb for Boutsko. Chromosomes 1, 2, and 3 had the largest number of adjacent SNPs in LD; in general, the total number of adjacent SNPs in LD tended to decrease with decreasing chromosome length (Figure S5). This, however, was not true for the Chios breed, which presented high numbers of SNPs in LD in chromosomes 7, 13, and 19. The rate of LD decay varied across chromosomes, suggesting stronger LD for certain quantitative trait loci (QTLs), indicative of the genetic structure and recombination dynamics within the sheep genome.

**Figure 7:**
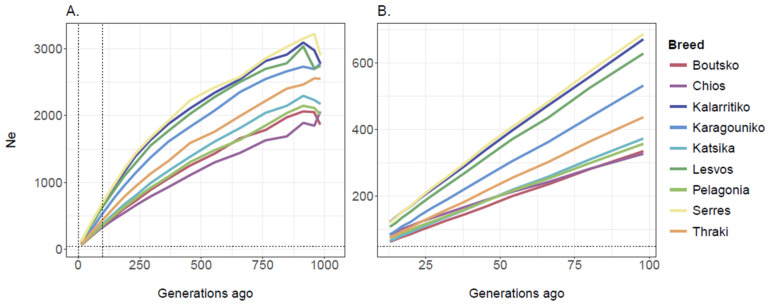
Effective population size (Ne) of Greek sheep breeds estimated over the last (**A**) 1000 and (**B**) 100 generations. The vertical dotted lines indicate the range from 0 to 100 generations ago while the horizontal dotted line represents Ne = 50.

Based on the 49,528 SNP dataset, ancestral Ne estimates were obtained for 27 distinct time points, corresponding to the past ~1000 generations for all breeds. Overall, the estimated Ne values revealed a declining trend over time. Across all generations, Serres, Kalarritiko, and Lesvos had a larger Ne compared to the other breeds. In the distant past, about 1000 generations ago, the Ne estimates were 2898 (Serres), 2777 (Karagouniko), 2773 (Kalarritiko), 2742 (Lesvos), 2548 (Thraki), 2173 (Katsika), 2059 (Chios), 2015 (Pelagonia), and 1866 (Boutsko) for the Greek breeds (Figure 7A). Yet, in the recent past, the obtained values for the last 100 generations were much lower; the Ne estimates, in increasing order, were 63 (Boutsko), 66 (Katsika), 74 (Pelagonia), 76 (Thraki), 84 (Chios), 85 (Karagouniko), 108 (Lesvos), 120 (Serres), and 122 (Kalarritiko) for the breeds, revealing a wider genetic pool for the Kalarritiko, Serres, and Lesvos breeds (Figure 7B).

## 4. Discussion

All species, whether a small bacterium or a higher animal, store enormous amounts of genetic information, which renders them unique and is essential for their reproduction, productivity, and survival. Even within species, the level of genetic diversity is remarkable, shaping, in the case of livestock, the development of distinct breeds worldwide. Sheep, over their evolutionary history, have evolved to withstand fluctuations in environmental conditions (shifts in oxygen levels, temperature, humidity, and water availability) and have managed to thrive in extreme climates, including high altitudes, heat, cold, and drought [44]. As different breeds thrive in specific environments, their preservation is of the utmost importance to ensure the long-term continuation of unique alleles. Within this context, we assessed the existing genetic diversity levels of the most numerous and frequently reared sheep breeds in Greece to explore their potential for long-term survival. Our findings revealed that Greek breeds maintain adequate levels of within-breed genetic diversity. This was supported mainly by the Ho and He levels, as well as inbreeding metrics and coefficients. Greek sheep breeds reported average Ho and He values of 0.356 and 0.361, respectively. Similar average He values were reported for other sheep breeds reared in countries in the Mediterranean basin, such as France (He: 0.354), Spain (average He: 0.346), and Italy (0.358) [24]. However, when looking at intensively reared breeds, such as East Friesian White (He = 0.290) [45], or threatened breeds, such as Karakachanska (Ho = 0.294) [25], the heterozygosity values are significantly lower, indicating genetic isolation or population bottlenecks [1]. Noteworthily, heterozygosity values are significantly higher when microsatellite markers are applied for the estimation of genetic diversity levels, since they mutate faster, rapidly generating new alleles through replication slippage [46]. Thus, the results across studies employing these different types of markers cannot be comparable, although they perform equally well for genetic diversity assessment [47]. Although limited to predefined loci, the use of a medium-density SNP array effectively captured the genetic diversity levels of Greek sheep breeds in our study, supporting their genetic differentiation. Recently, whole-genome sequencing (WGS) data for six Greek sheep breeds revealed approximately 21 million SNPs, highlighting their potential as reservoirs of genetic diversity [48]. This comprehensive catalog of genome-wide variants complements the SNP microarray-based assessment and offers a valuable resource for identifying novel, breed-specific genetic markers. Such markers can enhance the accuracy of population genetic analyses, improving genetic selection strategies and breeding programs tailored to Greek sheep populations.

The rich genetic resources of the nine Greek sheep breeds studied in our work were further supported by their resulting effective population sizes and LD patterns. Although all Greek breeds demonstrated steep declines over time, the current levels for all breeds remained above 63, which was the lowest Ne value, corresponding to the Boutsko breed. These values fall within the range posed by FAO for Ne values, namely between 50 and 100, to avoid short-term inbreeding depression phenomena and ensure the fitness of the population [49]. Yet, the term “short-term” is vague and could be interpreted subjectively. Furthermore, regarding the 50/500 rule initially proposed by Franklin (1980) [50], which suggests a minimum Ne of 500 to ensure long-term species adaptability, a lot of critical discussion has taken place among members of the scientific community, raising doubts and concerns regarding its use as an indicator of extinction risk [51]. Notably, Frankham et al. (2014) later suggested that these thresholds should be doubled to maintain evolutionary potential [52]. Thus, effective population sizes should be cautiously interpreted, taking into consideration other indicators, such as the LD decay patterns and inbreeding levels, before making decisions or classifying breeds as endangered or threatened, as, for example, knowledge of LD not only provides information about Ne but also provides insights into a population’s past demography [53]. Indeed, the rapid LD decay observed for Serres, Lesvos, and Kalarritiko verified the largest Ne values, suggesting a wider genetic diversity for these breeds. On the contrary, the Chios and Boutsko breeds presented higher *r*^2^ values over longer distances, informing on the selection pressure or bottleneck effects these breeds underwent. Yet, different evolutionary events contributed to this pattern; in the case of the Chios breed, the low genetic diversity indices and high *r*^2^ values confirm the selection pressure that this breed has been subjected to, whereas for the Boutsko breed, they could be indicators of population bottlenecks in their geographically isolated areas of origin. Such events can increase LD with some haplotypes being lost, increasing the effect of genetic drift [54]. This is evidenced for many breeds worldwide; for example, a recent study on sheep revealed that the Suffolk and Texel breeds demonstrated slower LD decay compared to the Belclare and Charollais breeds, likely due to increased genetic selection for production traits over time [55]. The prevailing trend indicates that local breeds subjected to less intensive breeding programs experience more rapid LD decay across distant markers, in contrast to cosmopolitan populations in which the LD extends for larger pairwise distances [53]. Research has demonstrated that sheep have lower LD than other livestock species due to less intensive genetic selection and descent from a larger gene pool [45].

In this respect, the Chios breed exhibited a high number of SNPs in LD for chromosomes 7, 13, and 19. These chromosomes enclose numerous QTLs related to milk production traits, suggesting a possible link between the observed LD and selection for dairy performance. In fact, according to the Animal QTL Database [56], several well-characterized SNPs located within these regions have been associated with milk traits. For example, OAR7_103688806 has been linked to milk yield, as well as fat and protein percentages. Additional SNPs associated with the milk fat percentage included s53991.1, DU404838_401.1, s06902.1, OAR19_11957218.1, OAR19_41862754.1, OAR19_42652210.1, OAR19_56538781_X.1, and s01256.1. SNPs related to protein yield included OAR7_16477111.1, OAR7_17669851.1, and OAR7_102675896.1, while SNPs associated with milk yield included OAR7_16477111.1, OAR7_18924134.1, OAR7_15000911.1, OAR13_29033933.1, s72187.1, and OAR19_11957218.1 [57]. All these SNPs were found in high LD for the Chios breed. The presence of such markers in high LD suggests that selection for milk production traits within national breeding programs has probably shaped the genomic landscape of the Chios breed.

As inbreeding levels provide information on a population’s past genetic events and demographic evolution over time, we further estimated several homozygosity indices. However, there is a lack of standards for ROH definition across studies, which increases the likelihood of biased and false-positive results [58]. Hence, in our study, we applied the guidelines proposed by Meyermans et al. (2020) [23] for an adequate and robust ROH analysis using medium-density SNP data, reporting all relative parameters to substantially improve the overall quality and uniformity of ROH analyses. Thus, ROH detection was performed without MAF and LD filtering, as such SNP filtering does not improve ROH detection, and, additionally, impacts the detection of large ROHs [23]. It should also be noted that the density of the microarray applied is crucial, since medium-sized arrays lack the sensitivity to precisely determine small segments, while high-density arrays fail to disclose certain ROH patterns [59]. Based on our findings, the nine Greek breeds demonstrate relatively modest levels of autozygosity, as evidenced by ROH segments, in addition to F_ROH_, F_GRM_, and F_IS_. Overall, high numbers of short ROHs (1–5 Mb) were observed for Greek sheep breeds, suggesting that ancient relatedness occurred many generations ago or recent admixture and, thus, recombination events that could result in the breakdown of larger ROHs [60,61]. On the contrary, long ROHs (over 10 Mb) were the least prevalent, suggesting recent inbreeding and poor breeding management practices in the studied populations. Apart from the Chios breed, which presented the highest levels of inbreeding (F_IS_, F_ROH_, and F_GRM_), all the other Greek breeds did not significantly vary regarding autozygosity levels. This is indicative of the breeders’ breeding management strategies, regardless of the rearing system or farm geographic location. Notably, the results of all inbreeding indices revealed high standard deviation values, suggesting the high variability in autozygosity levels within the breeds. For Chios, the increased autozygosity was expected, as it is the only breed subjected to intense selection over the last decades through its participation in genetic improvement programs. However, although increased F_ROH_ values reflect the significant impact of fitness and a reduction in the performance of offspring in sheep [62,63], the inbreeding values obtained for the Chios breed do not suggest that this breed could suffer from inbreeding depression and the purging of deleterious alleles. Consistent with our results, the F_ROH_ values for Italian breeds ranged from 0.016 in Comisana sheep to 0.099 in Valle del Belice sheep [64]. In our study, the estimation of homozygosity using the F_PED_ index with pedigree data was not feasible, since the relevant records were not available from the farmers. However, the information obtained using this coefficient is questioned, as it may underestimate the genome homozygosity in sheep [65].

The genetic relatedness of Greek breeds and the levels of their genetic differentiation were investigated using the F_ST_ index. The most differentiated breed pair was Chios and Boutsko (F_ST_ = 0.157), which was expected, as their origin and breeding purposes are substantially different. The Chios breed, originating from Anatolia and influenced by the Ruda sheep type, is reared under extensive breeding systems towards milk production. On the other hand, the Boutsko breed can be found in semi-mountainous or mountainous areas in Western Macedonia, Epirus Municipality, or alongside the Pindus mountain range and is bred both for meat and milk, mainly supporting local, traditional communities. Furthermore, low genetic differentiation was found between the mainland Serres breed and the Karagouniko (F_ST_ = 0.050), Kalarritiko (F_ST_ = 0.055), and Lesvos (F_ST_ = 0.055) breeds. However, the low genetic differentiation between Serres and Kalarritiko is not in agreement with the PCA results or the ML dendrogram clustering, and, additionally, the geographic region of origin, breeding systems, and management practices of these two breeds differ significantly. For sheep, an F_ST_ range between 0.06 and 0.17 could be adopted for breed differentiation; however, the use of these values as benchmarks must be conditional on acknowledging their stochastic nature and probable dependence on geographic factors [66]. In fact, it was recently reported that the sole use of the F_ST_ index for capturing the genetic differentiation between breed pairs has shortcomings that complicate the assessment of population differentiation, not necessarily measuring the most meaningful aspects in population genetics [67]. Thus, the F_ST_ index results’ interpretation should be complemented with other genetic distance calculations, multivariate analysis, and admixture plots, ideally within a broader context of landscape genomics.

From an evolutionary aspect, our findings verified that the nine Greek sheep breeds were clustered into two major groups when analyzed along with other, international breeds; one of them was an independent group involving Chios and Sakiz breeds and the other one grouped all the Greek mainland breeds and clustered them with those of Eastern Europe. Gene flow analysis highlighted that the Chios breed was in very close proximity to the Turkish Sakiz breed, supported by bootstrap values higher than 90%. The name “Sakiz” is the Turkish name for the Greek island of Chios in the Aegean Sea, and historical data support that Sakiz sheep were probably brought to Çesme, a town in the province of Izmir, from the Greeks many decades ago [68]. Thereafter, Chios influenced other Greek sheep breeds like Karagouniko, Serres, Pelagonia (Figure S6), and Kymi, but no contribution of genes to other European or African breeds was detected, based on the seven identified past migration events inferred in our work. This was also highlighted by another study that included a different genotyping dataset for Chios; only at m = 20 was a medium-weight migration edge traced, linking Chios to Cyprus fat-tailed sheep [1]. Gene flow studies involving Greek sheep are limited, with only a few insular breeds analyzed to date [1]. Ciani et al. (2020) estimated the gene flow patterns of insular Greek breeds, yet the influence of the Zackel-type sheep in Greek lowland breeds remained unexplored. Moreover, in their study, the sample size for the Lesvos and Kymi breeds was particularly small (N = 6 for each breed), which may have affected the accuracy and reliability of the results [69]. Hence, in our work, we aimed to address this gap. Regarding the lowland and mountainous Greek breeds, it was evidenced that they were strongly influenced by the Eastern European countries and the Balkans. Katsika and Karagouniko were mainly influenced by the Tsigaia sheep originating from the Carpathian Mountains. In fact, the close proximity of Carpathian and Greek livestock populations was also evidenced for the Eghoria Greek goat breed [70], highlighting the historically common management practices applied and the joint rearing of sheep and goats. Regarding the geographically isolated populations of the Epirus mountain range, Kalarritiko and Boutsko exhibited stronger genetic relatedness with breeds from the Southern Balkans, specifically Albania and Bulgaria. In particular, the close genetic relationship with the Albanian Shkodrane sheep can be attributed to historical (often trans-border) transhumance routes, shared cultural and historical community ties, and similar breeding environments. Our findings cast doubt on the common origin of the Kalarritiko and Comisana breeds reported by the Greek Ministry of Rural Development and Food [18], since neither their genetic clustering nor the inferred migration patterns support this. Additionally, our results support a genetic relatedness between the Kalarritiko and Boutsko breeds, indicating a shared genetic background. However, the Boutsko populations appear to have undergone stronger genetic drift, likely due to random mating, longer isolation, or population bottlenecks, as a higher drift value was obtained for this breed (Figure S6). Furthermore, gene flow analysis among Greek sheep breeds highlighted the Chios breed’s dominant role as a source of genetic improvement for milk traits, impacting breeds such as Serres, Karagouniko, and Pelagonia. Such crossbreeding practices are common among Greek sheep breeders, primarily intended to enhance milk production by incorporating high-yielding breeds such as Chios. At the same time, there is an effort to preserve important traits from other indigenous breeds, such as resilience to harsh environments, resistance to diseases, and adaptability, given that the Chios breed is known to be more susceptible to extensive breeding systems [21,71]. Therefore, preserving local breeds and their levels of genetic diversity is crucial for sustaining these breeding strategies, as such genetic resources underpin the long-term adaptability and resilience of sheep populations in the face of environmental change and production challenges.

## 5. Conclusions

In this study, we present the first large-scale evaluation of the genetic resources of nine indigenous Greek sheep breeds. As genetic erosion threatens many indigenous breeds, the incorporation of genetic tools into animal breeding, genetic improvement, and breed conservation programs can contribute towards conserving biodiversity. In our work, we found that Greek breeds maintain adequate levels of genetic diversity, without signs of excessive inbreeding. Chios exhibited the lowest within-breed genetic diversity levels and was the most differentiated breed. Following Chios, the Lesvos breed was also found to be genetically differentiated from the rest of the mainland breeds. Moreover, we revealed the genetic proximity between the Serres and Karagouniko breeds, mostly attributed to management practices. In addition, the genetic similarity between the Boutsko and Kalarritiko breeds was confirmed. Furthermore, with our work, we provide novel insights into the westward migration waves of sheep, which, for the first time, involved the Greek territory. We revealed the genetic introgression of Greek insular breeds with breeds from Anatolia and the transfer of genetic material from breeds of Eastern Europe to lowland Greek sheep breeds. Our results support the application of genomic approaches for understanding the population genetic structure, assessing inbreeding levels, and monitoring crossbreeding practices within sheep flocks. In addition, our findings contribute towards the proper design of reproductive breeding schemes by offering valuable insights into genetic improvement and reproduction management; through the genetic characterization and selection of individuals, such efforts can be designed to also minimize the loss of genetic diversity. Ultimately, these approaches will increase adaptability to environmental changes and reduce susceptibility to diseases, while also promoting the long-term viability of each breed. Our work forms the basis on which effective genetic improvement and selection schemes can operate in Greece, enabling the production of breeding stock or animal genotypes with desired traits.

## Figures and Tables

**Table 1:** Genetic differentiation of Greek sheep breeds as measured by Wright’s pairwise fixation index (F_ST_).

Breed	Boutsko	Chios	Kalarritiko	Karagouniko	Katsika	Lesvos	Pelagonia	Serres
**Chios**	0.157							
**Kalarritiko**	0.070	0.120						
**Karagouniko**	0.095	0.095	0.060					
**Katsika**	0.109	0.127	0.074	0.071				
**Lesvos**	0.100	0.089	0.067	0.060	0.079			
**Pelagonia**	0.128	0.139	0.093	0.090	0.108	0.096		
**Serres**	0.086	0.089	0.055	0.050	0.068	0.055	0.084	
**Thraki**	0.108	0.119	0.073	0.071	0.087	0.072	0.106	0.063

**Table 2:** Within-breed genetic diversity indices based on the 49,528 filtered single nucleotide polymorphisms (SNPs). N_P%_: Percentage of polymorphic loci; Ho: observed heterozygosity; He: expected heterozygosity; π: nucleotide diversity; SD: standard deviation.

Breed	N_P%_	Ho	He	π
		Mean ± SD	Mean ± SD	Mean ± SD
Boutsko	94.19%	0.352 ± 0.167	0.353 ± 0.143	0.338 ± 0.162
Chios	93.17%	0.326 ± 0.164	0.344 ± 0.146	0.327 ± 0.157
Kalarritiko	97.89%	0.366 ± 0.147	0.367 ± 0.134	0.363 ± 0.173
Karagouniko	97.73%	0.379 ± 0.155	0.373 ± 0.130	0.371 ± 0.178
Katsika	95.02%	0.353 ± 0.162	0.363 ± 0.136	0.353 ± 0.171
Lesvos	98.03%	0.369 ± 0.151	0.366 ± 0.134	0.363 ± 0.173
Pelagonia	94.82%	0.348 ± 0.159	0.355 ± 0.141	0.341 ± 0.163
Serres	98.81%	0.357 ± 0.142	0.370 ± 0.131	0.369 ± 0.176
Thraki	95.82%	0.357 ± 0.152	0.366 ± 0.134	0.357 ± 0.171

**Table 3:** Average inbreeding coefficient values and standard deviation (SD) per breed. F_IS_: Wright’s inbreeding coefficient; F_GRM_: Genomic relationship matrices inbreeding coefficient; F_ROH_: Runs of homozygosity inbreeding coefficient.

Breed	F_IS_	F_GRM_	F_ROH_
	Mean ± SD	Mean ± SD	Mean ± SD
Boutsko	0.082 ± 0.068	0.068 ± 0.067	0.059 ± 0.048
Chios	0.148 ± 0.035	0.126 ± 0.036	0.082 ± 0.027
Kalarritiko	0.043 ± 0.042	0.040 ± 0.041	0.030 ± 0.031
Karagouniko	0.011 ± 0.050	0.036 ± 0.052	0.019 ± 0.036
Katsika	0.077 ± 0.038	0.083 ± 0.040	0.062 ± 0.031
Lesvos	0.037 ± 0.041	0.038 ± 0.037	0.026 ± 0.031
Pelagonia	0.090 ± 0.042	0.079 ± 0.042	0.068 ± 0.030
Serres	0.068 ± 0.061	0.072 ± 0.056	0.056 ± 0.046
Thraki	0.066 ± 0.060	0.072 ± 0.059	0.056 ± 0.047

## Data Availability

The data presented in this study are available on request from the corresponding author, due to confidentiality agreements.

## Supplementary Materials

The following supporting information can be downloaded at: https://www.mdpi.com/article/10.3390/biology14070845/s1, Figure S1: Images of individuals or flocks for the nine indigenous Greek sheep breeds included in the study. (A) Boutsko, (B) Chios, (C) Kalarritiko, (D) Karagouniko, (E) Katsika, (F) Lesvos, (G) Pelagonia, (H) Serres, (I) Thraki; Table S1: Morphological characteristics of the nine indigenous Greek sheep breeds; Table S2: Number of individuals sampled per breed, theoretical/historical region of origin, location of farms that participated in the study, and breeding systems applied for each breed in the various farms; Table S3: Information on breeds and populations included in the analyses; Figure S2: TreeMix analysis in 54 sheep populations with seven migration events. Nodes’ robustness was estimated with 100 bootstrap replicates. Only bootstrap values above 50% are shown. Migration edges are colored according to their migration weight. Breeds are colored according to their geographical origin (brown for Africa, green for Asia, dark green for the Balkans and Eastern Europe, blue for Greece, and red for Southern and Western Europe); Figure S3: Total number and length in Megabases (Mb) of runs of homozygosity (ROHs) per animal. Different colors indicate different breeds; Figure S4: Distribution of Runs of Homozygosity (ROHs) on autosomes. Each bar represents the total number of ROHs per chromosome. The red line shows the average percentage of ROHs for each chromosome; Figure S5: Distribution of Single Nucleotide Polymorphisms (SNPs) in Linkage Disequilibrium (LD) per chromosome for all breeds; Figure S6: Treemix analysis with two migration events for the Greek breeds.

## Abbreviations

The following abbreviations are used in this manuscript:

DNA	Deoxyribonucleic acid
FIS	Wright’s inbreeding coefficient—Individual within Subpopulation
FST	Wright’s inbreeding coefficient—Subpopulation within Total population
HWE	Hardy-Weinberg Equilibrium
K2EDTA	Potassium ethylenediaminetetraacetic acid
kb	Kilobase
LD	Linkage Disequilibrium
MAF	Minor Allele Frequency
Mb	Megabase
Ne	Effective population size
PCA	Principal Component Analysis
ROH	Runs of homozygosity
SNP	Single Nucleotide Polymorphism
QTLs	Quantitative Trait Loci
