# Supplementary material for: Genetic Diversity, Population Structure, and Historical Gene Flow Patterns of Nine Indigenous Greek Sheep Breeds
Source: Biology (Basel). 2025 Jul 10;14(7):845. doi: 10.3390/biology14070845 (PMC12292511; doi:10.3390/biology14070845)
Supplement: Supplementary file 1 [file biology-14-00845-s001.zip › S1 Table.pdf]

Table S1. Morphological characteristics of the nine indigenous Greek sheep breeds [6,14,18].

Breed	Color	Height (cm)		Weight (kg)		Ears	Nose profile (rostrum)	Tail
		Male	Female	Male	Female			
Boutsko	Completely white, black, reddish-brown or spotted	55 - 70	30 - 50	40 - 70	53 - 65	Narrow, medium-sized, almost horizontal	Curved	Long and thin
Chios	White with black spots around the eyes, ears, nose, belly, and limbs; often, the entire face is black	79 - 84	70 - 76	65 - 90	50 - 70	Big, horizontal or slightly pendulous	Slightly curved	Semi-fat
Kalarritiko	White face with cheeks, ears, nasal tip, and frequently the distal extremities of the limbs predominantly displaying a deep red or pale pigmentation; forehead and nasal dorsum are typically white	65 - 68	57 - 60	60 - 65	42 - 46	Narrow, medium-sized, almost horizontal	Slightly curved	Semi-long and thin
Karagouniko	Typically, completely white with black spots on the face and limbs, rarely total black	66 - 77	55 - 68	65 - 80	45 - 60	Big, horizontal or slightly pendulous	Curved	Small to medium length and thin
Katsika	White with black spots around the eyes, ears, mouth, and cheeks	68 - 72	58 - 62	65 - 72	47 - 55	Narrow, medium-sized, almost horizontal	Curved	Long and thin
Lesvos	White with black or brown spots on the nose and limbs.	67 - 72	60 - 65	60 - 70	42 - 50	Small and pendulous	Slightly curved	Very long, semi-fat

	Occasionally, entirely black, dark brown, or variegated brown shades							
Pelagonia	Mainly white. Facial color ranges from total white to white with black spots around the eyes and nose	72 - 75	62 - 66	68 - 76	55 - 60	Medium-sized, almost horizontal	Slightly curved	Long and thin
Serres	White with black spots on the head and limbs or totally black	70 - 75	65 - 67	65 - 80	45 - 62	Thin, medium-sized, horizontal or slightly pendulous	Curved	Medium length and thin
Thraki	White with black or brown spots on the face, the limbs, and around the eyes; sometimes brown with black face and limbs	62 - 65	55 - 60	45 - 55	35 - 45	Narrow, medium-sized, almost horizontal	Straight	Long and thin
