# Supplementary material for: Genetic Diversity, Population Structure, and Historical Gene Flow Patterns of Nine Indigenous Greek Sheep Breeds
Source: Biology (Basel). 2025 Jul 10;14(7):845. doi: 10.3390/biology14070845 (PMC12292511; doi:10.3390/biology14070845)
Supplement: Supplementary file 1 [file biology-14-00845-s001.zip › S2 Table.pdf]

Table S2. Number of individuals sampled per breed, theoretical/historical region of origin, location of farms that participated in the study, and breeding systems applied for each breed in the various farms.

Breed	Number of individuals	Breed origin	Farm location	Breeding system
Boutsko	29	Epirus	Trikala and Ioannina	Semi-extensive, transhumance
Chios	24	Chios	Halkidiki	Intensive
Kalarritiko	46	Epirus	Ioannina	Extensive, transhumance
Karagouniko	26	Thessaly	Karditsa	Intensive
Katsika	20	Epirus	Ioannina	Semi-extensive, transhumance
Lesvos	39	Lesvos	Kilkis	Semi-intensive
Pelagonia	36	Western Macedonia	Florina	Intensive
Serres	42	Serres	Serres	Semi-intensive
Thraki	30	Thraki	Evros	Semi-extensive
