# Supplementary figures and images for: Genetic Diversity, Population Structure, and Historical Gene Flow Patterns of Nine Indigenous Greek Sheep Breeds
Source: Biology (Basel). 2025 Jul 10;14(7):845. doi: 10.3390/biology14070845 (PMC12292511; doi:10.3390/biology14070845)

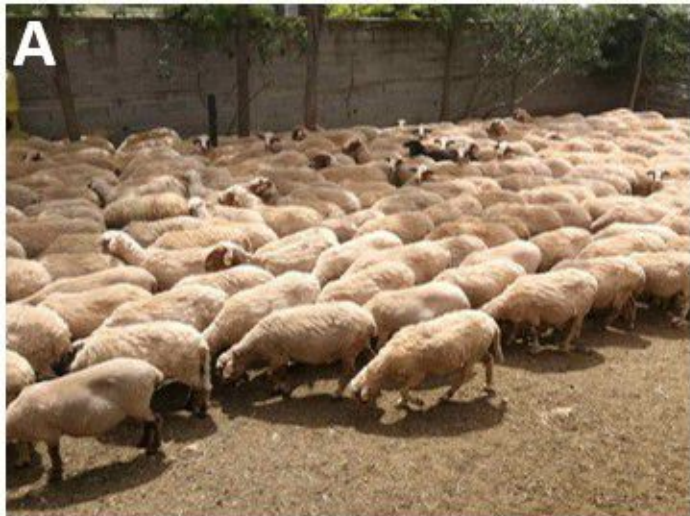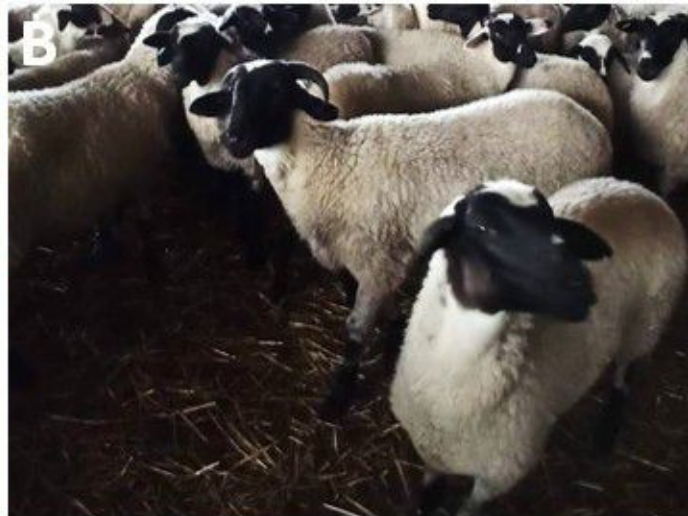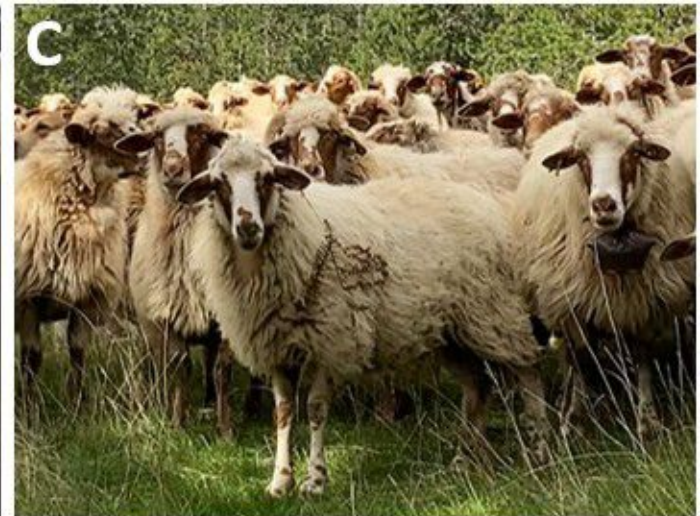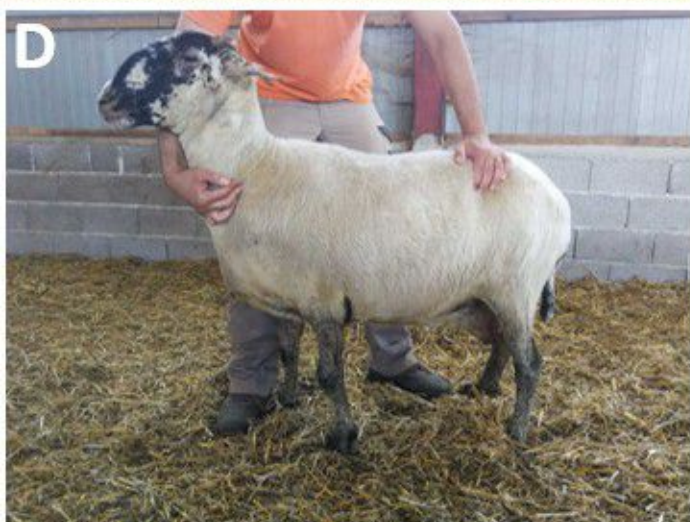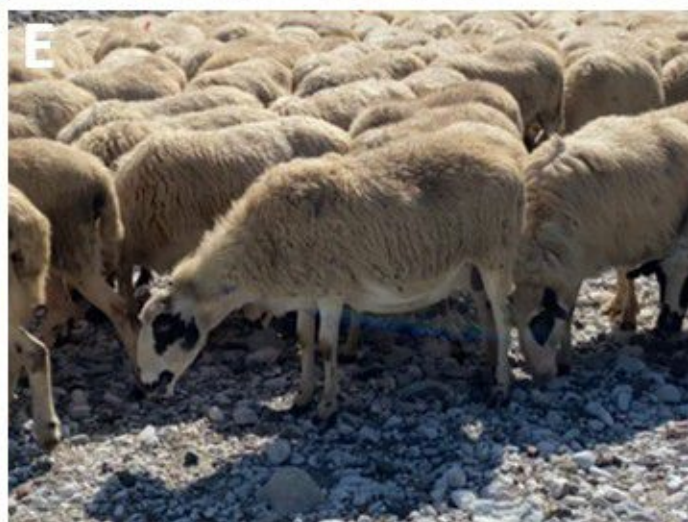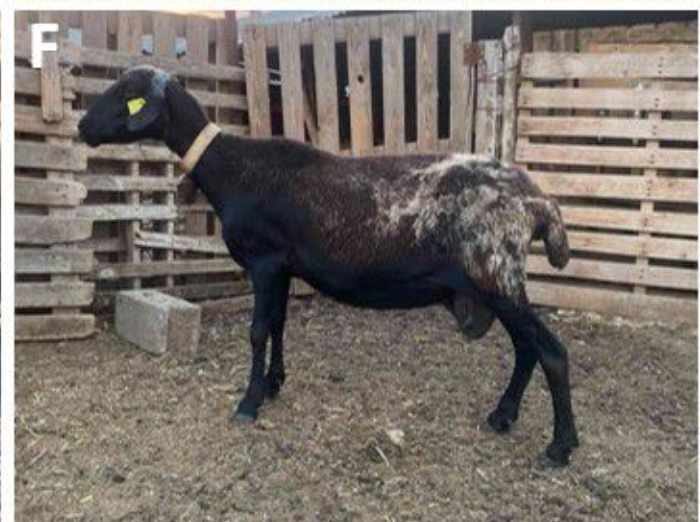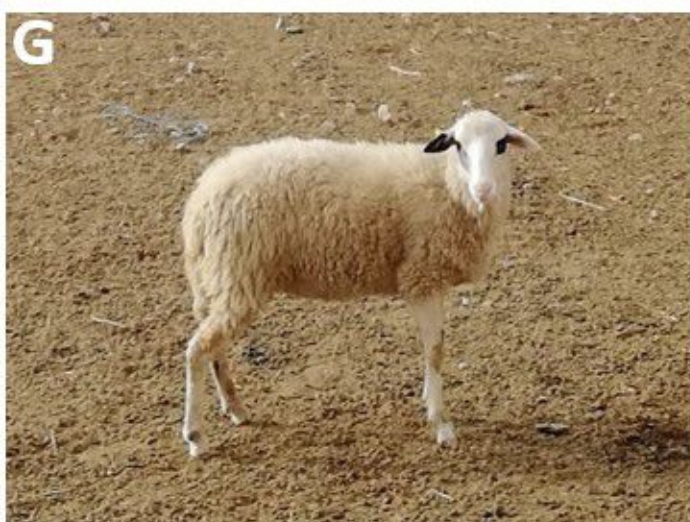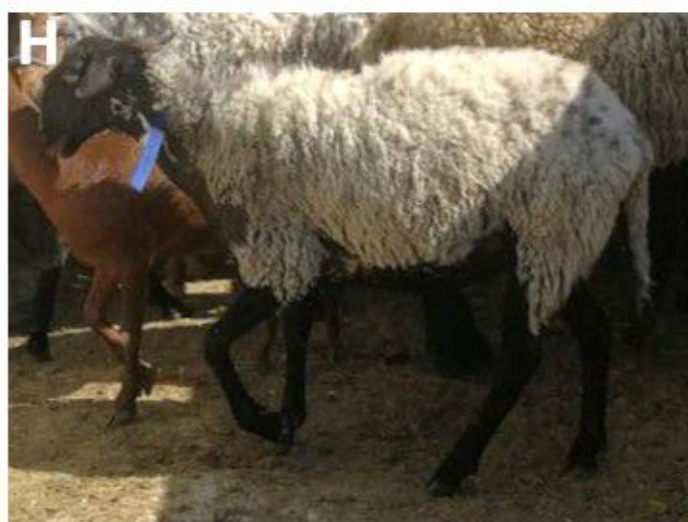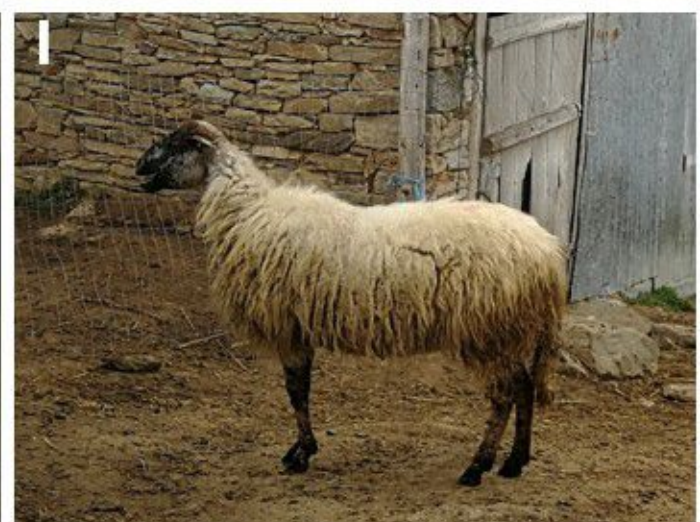

Supplement: Supplementary file 1 [file biology-14-00845-s001.zip › S1 Fig.pdf]

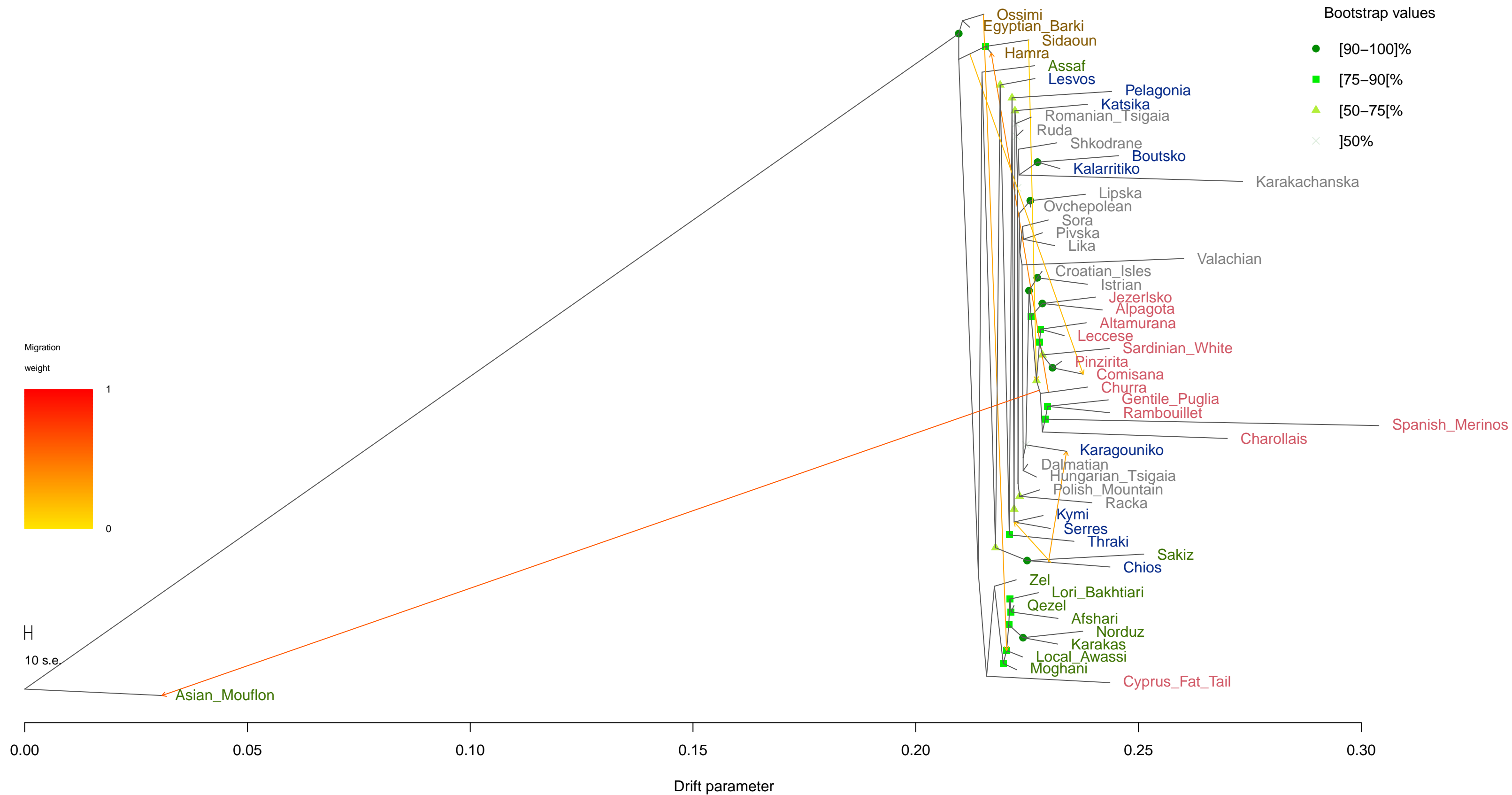

Supplement: Supplementary file 1 [file biology-14-00845-s001.zip › S2 Fig.pdf]

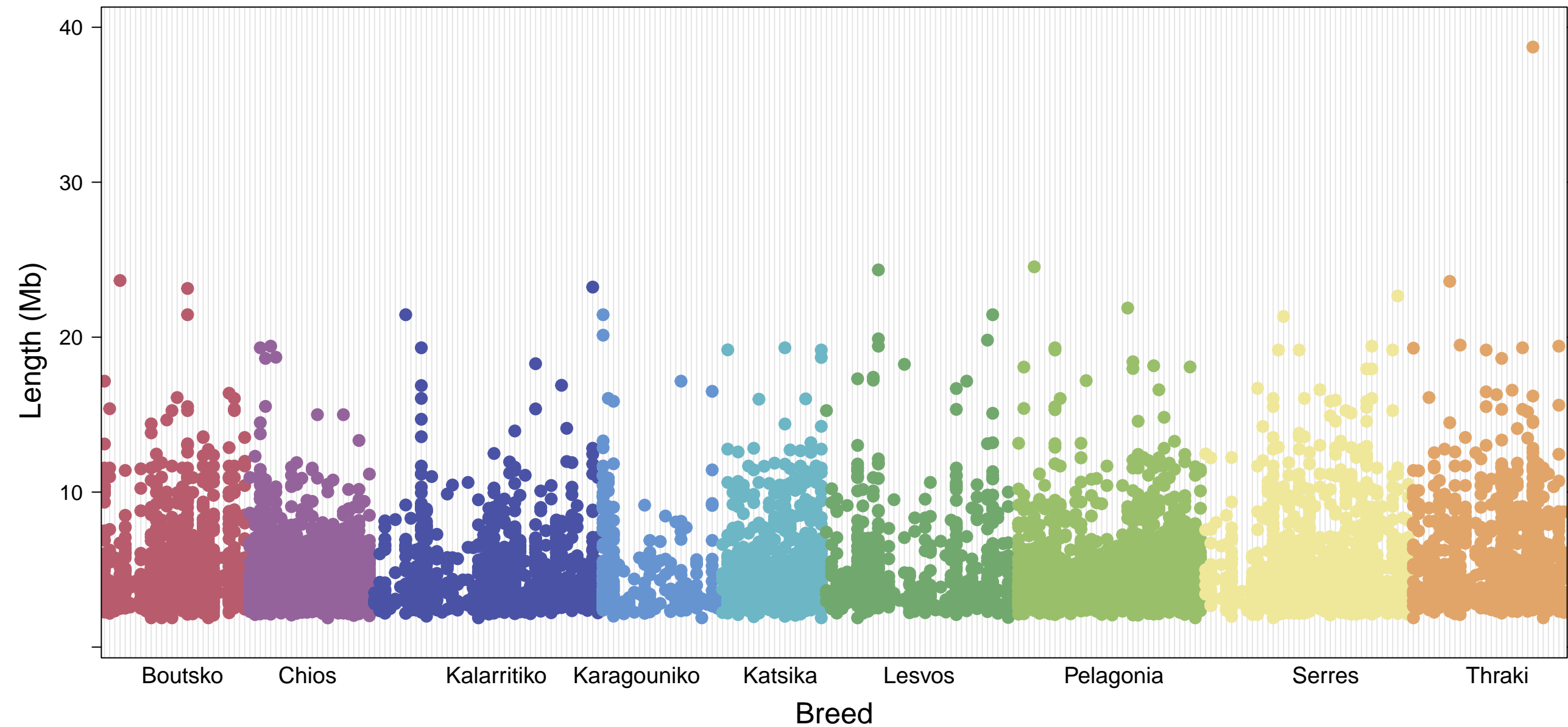

Supplement: Supplementary file 1 [file biology-14-00845-s001.zip › S3 Fig.pdf]

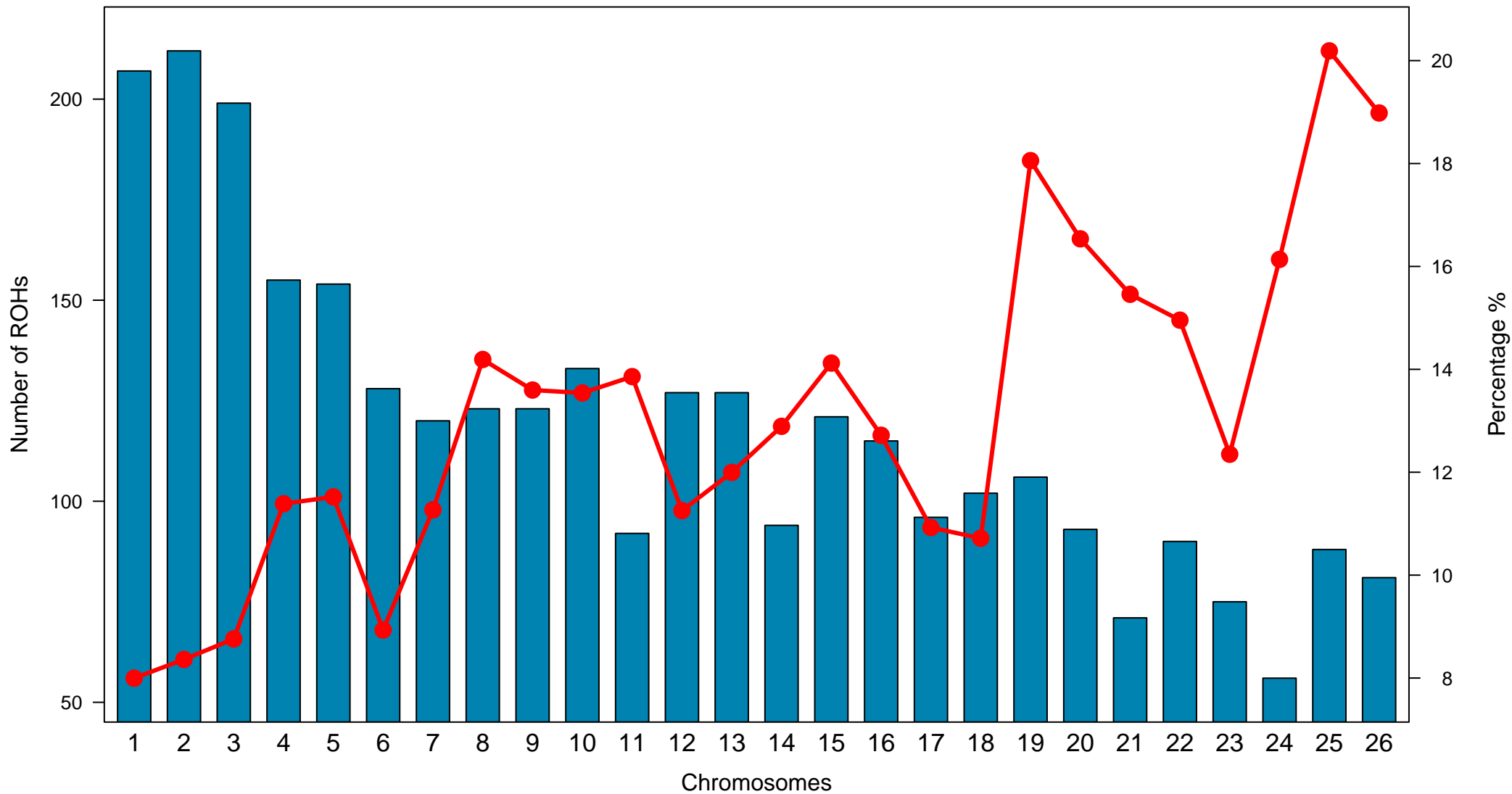

Supplement: Supplementary file 1 [file biology-14-00845-s001.zip › S4 Fig.pdf]

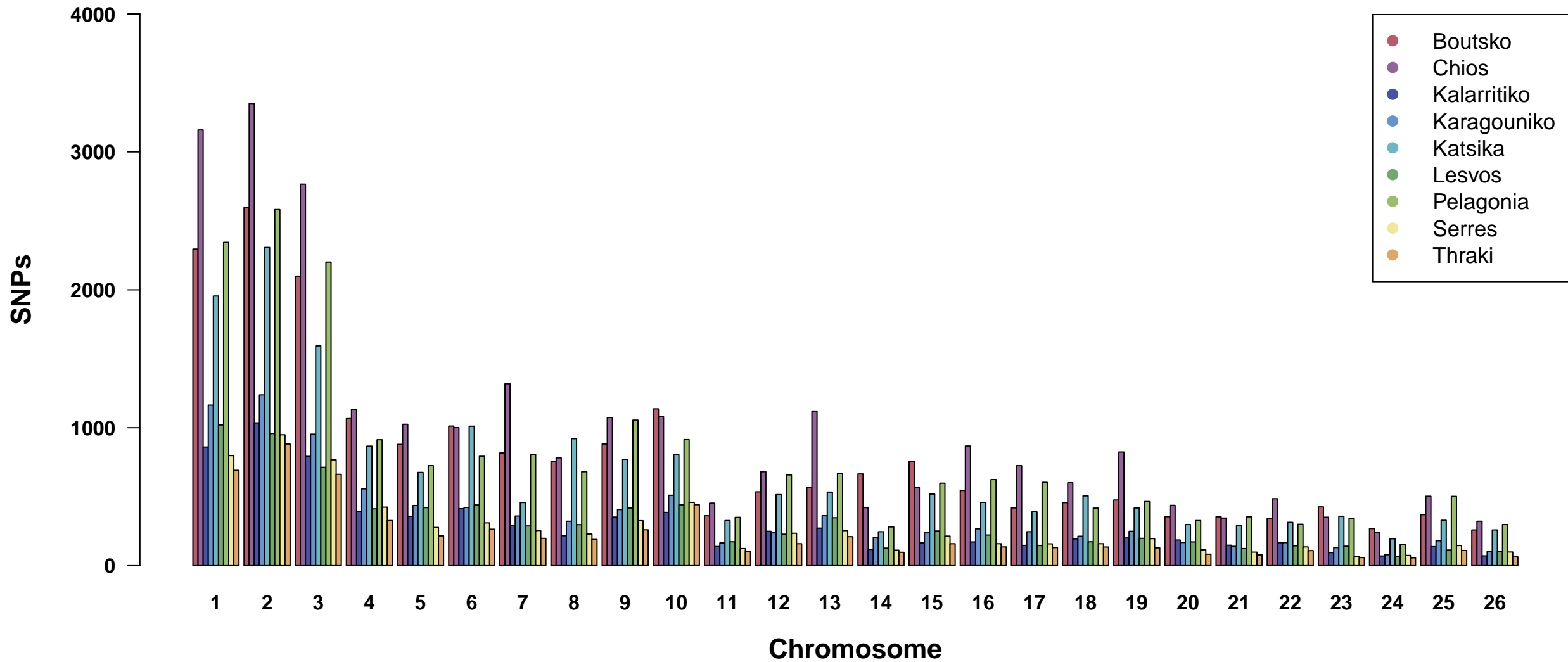

Supplement: Supplementary file 1 [file biology-14-00845-s001.zip › S5 Fig.pdf]

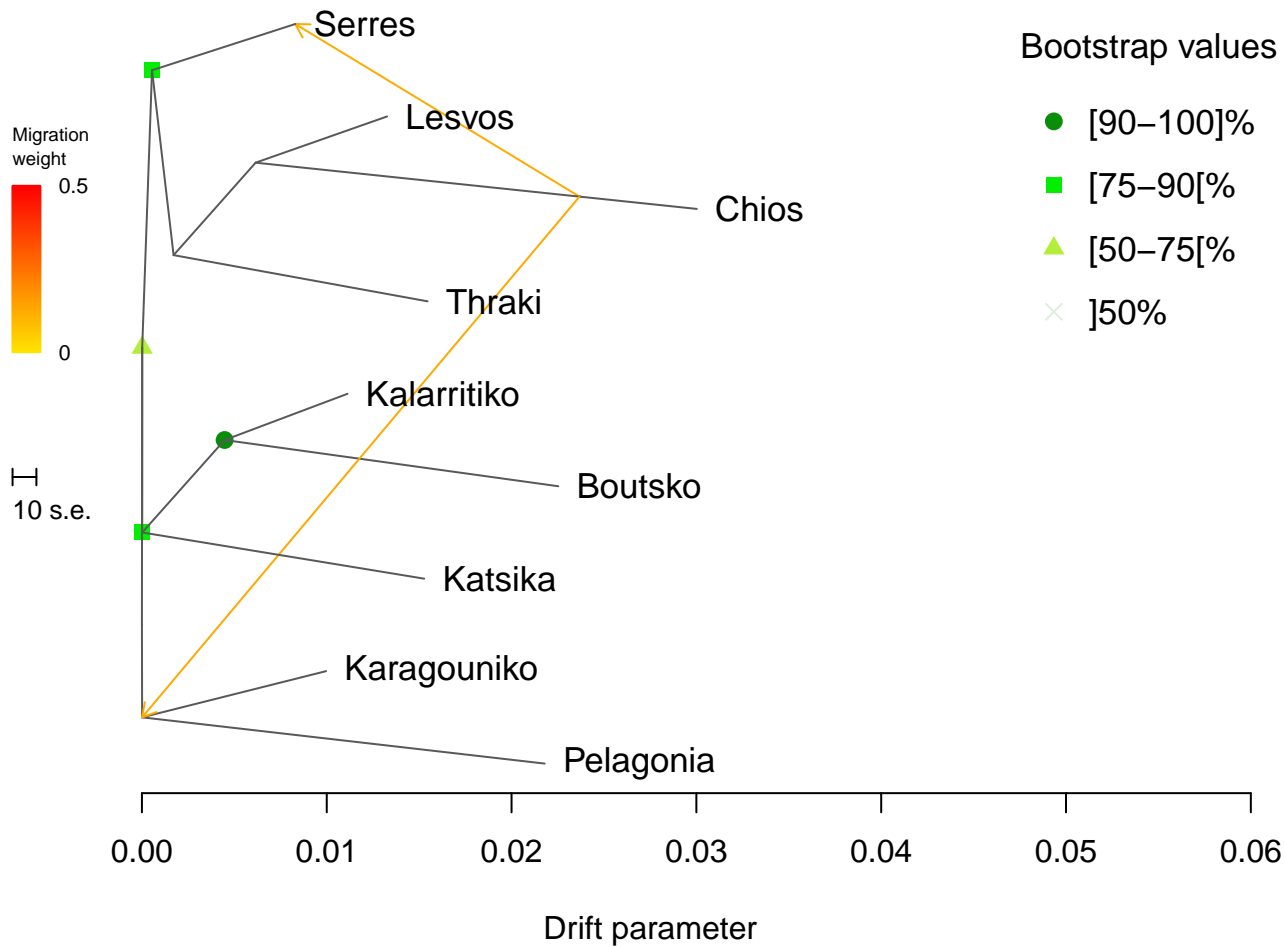

Supplement: Supplementary file 1 [file biology-14-00845-s001.zip › S6 Fig.pdf]
